# Supplementary material for: Mumefural Improves Recognition Memory and Alters ERK-CREB-BDNF Signaling in a Mouse Model of Chronic Cerebral Hypoperfusion
Source: Nutrients. 2023 Jul 24;15(14):3271. doi: 10.3390/nu15143271 (PMC10383324; doi:10.3390/nu15143271)
Supplement: Supplementary file 1 [file nutrients-15-03271-s001.zip › nutrients-2512814-supplementary.pdf]

Supplementary materials and methods

Protein digestion

Tissues were cryopulverized with liquid nitrogen using the Covaris Tissue CryoPrep System (Covaris, Woburn, MA, USA). Powdered samples were collected and placed in glass tubes. The samples were suspended in lysis buffer (8 M urea–0.1 M Tris-HCl buffer, pH 8.5) and protease inhibitor cocktail, followed by sonication for 20 min at 15°C using Covaris S2 Focused-Ultrasonicator (Covaris, Woburn, MA, USA). Protein concentration was quantified using the Pierce BCA Protein Assay Kit (Thermo Fisher Scientific). The digestion step was performed using FASP on a Microcon 30K centrifugal filter device (Millipore, Billerica, MA, USA). Each 100 μg of protein sample was reduced by incubation with 5 mM tris(2-carboxyethyl)phosphine (TCEP) at 37°C for 30 minutes and alkylated with 50 mM iodoacetic acid (IAA) at 25°C for 1 hour in the dark. After sequential washing with lysis buffer and 50 mM ammonium bicarbonate (ABC), the proteins were digested with trypsin (enzyme to protein ratio of 1:50; w/w) at 37°C for 18 h. The resulting peptide mixtures were collected in new tubes, and trypsin was inactivated by acidification with 15 μL of formic acid. The digested peptides were desalted using C18 spin columns (Harvard Apparatus, Holliston, MA, USA), and the peptides were eluted with 80% acetonitrile in 0.1% formic acid (Honeywell, Charlotte, NC, USA) in water.

LC-MS/MS analysis

The prepared samples were analyzed using a Q-Exactive Orbitrap hybrid mass spectrometer (Thermo Fisher Scientific, Waltham, MA, USA) coupled to a nanoACQUITY UPLC system (Waters, Milford, USA). We used a 3 cm x 150 μm ID trapping column packed with 3 μm C18 resin and a 100 cm x 75 μm ID analytical column packed with 3 μm C18 resin for peptides, depending on the hydrophobicity of the peptides. The mobile phase solvents were (A) 0.1% formic acid in water and (B) 0.1% formic acid in 90% acetonitrile, and the flow rate was set at 300 nL/min. The gradient of mobile phase was as follows: 10–40% solvent B in 160 min, 40–80% solvent B in 5 min, holding at 80% of solvent B in 13 min, 80–10% solvent B in 0.1 min, and 4% solvent B for 1.9 min. A data dependent acquisition method was used and the top 10 precursor peaks were selected and isolated for fragmentation. Ions were scanned at high resolution (70,000 in MS1, 17,500 in MS2 at m/z 400) and the MS scan range was 400-2,000 m/z in both the MS1 and MS2 levels. Precursor ions were fragmented with NCE (Normalized Collisional Energy) 30%. Dynamic exclusion was set to 30 seconds.

Proteome data analysis

Protein quantification was performed using MaxQuant version 1.5.8.3 (www.coxdocs.org) with the Andromeda search engine. The parameters were set as follows: maximum of two missed trypsin cleavages, 20 ppm for first search peptide tolerance, 4.5 ppm for main search peptide tolerance, and an FDR cutoff of 1%. Fixed and variable modifications: static carbamidomethylation of cysteine; and variable modifications including oxidation (methionine, +15.995 Da), carbamylation (protein N-terminal, +43.0006 Da). Data were analyzed and visualized using Mass Profiler Professional version 12.6.1 (Agilent Technologies, Santa Clara, CA, USA).

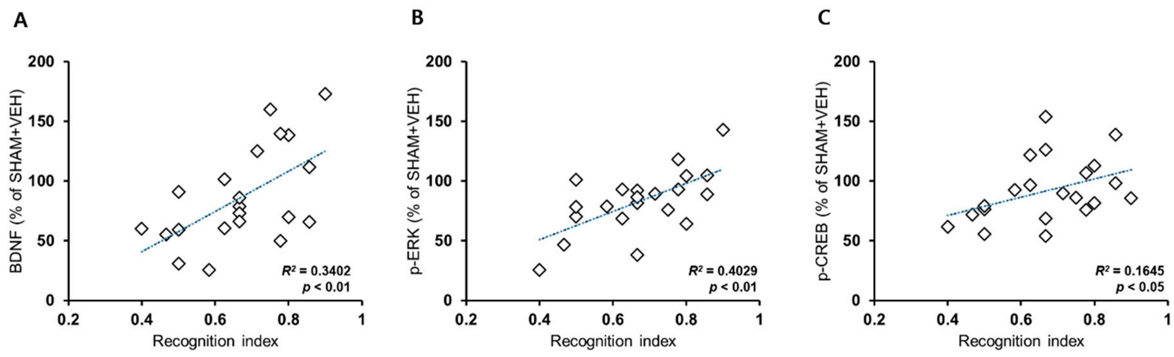

Supplementary Figure S1. Correlation between object recognition memory and ERK/CREB/BDNF signaling. Correlation between recognition index in the novel object recognition test and levels of hippocampal BDNF **(A)**, phosphorylated ERK **(B)**, and phosphorylated CREB **(C)**. Each point represents an individual sample. All correlation tests were performed using Pearson´s correlation analysis.

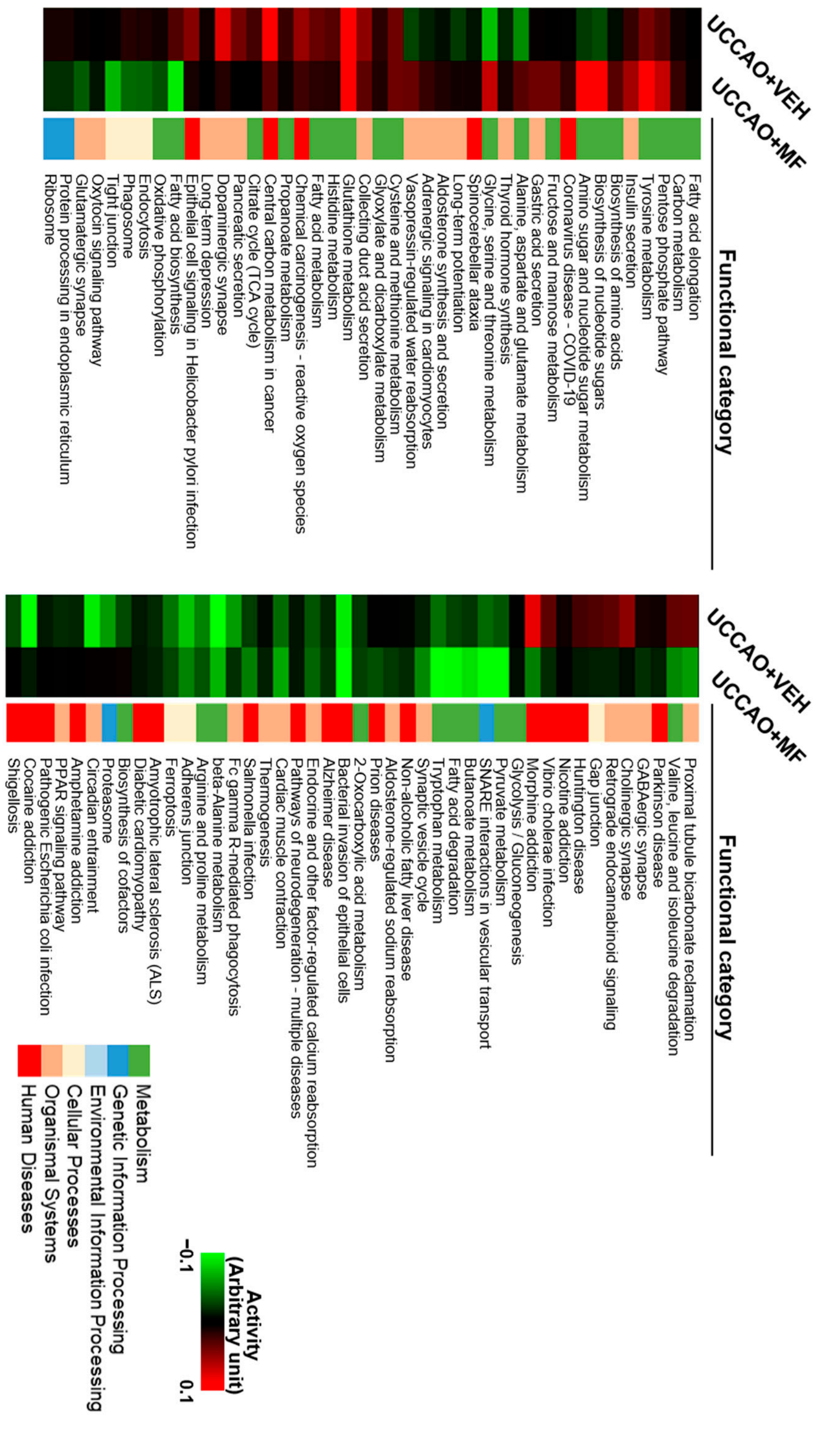

Supplementary Figure S2. Analysis of pathway activity. Pathways with a p-value <0.001 in the sample were selected. As shown in the scale bar at the bottom, pathways with increased or decreased activity levels were indicated in red or green, respectively. As indicated in the right panel, individual pathways were divided into six categories according to their function.

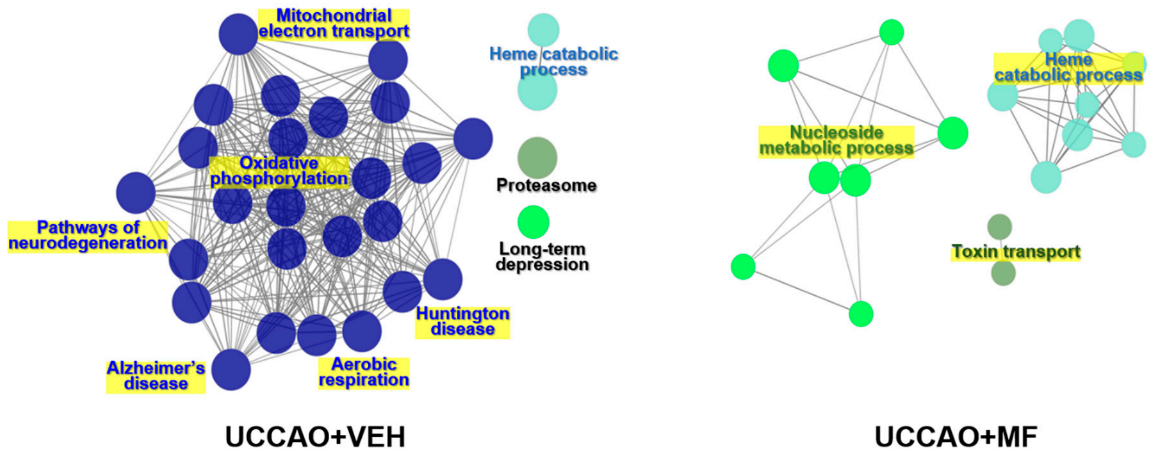

Supplementary Figure S3. GO-term enrichment analysis. Selected GO terms are displayed in network format. Individual nodes represent each GO term, and their size represents the number of proteins contained in the node. Nodes that are functionally closely related are marked with the same color, and selected representative functions are highlighted.

Figure 5A

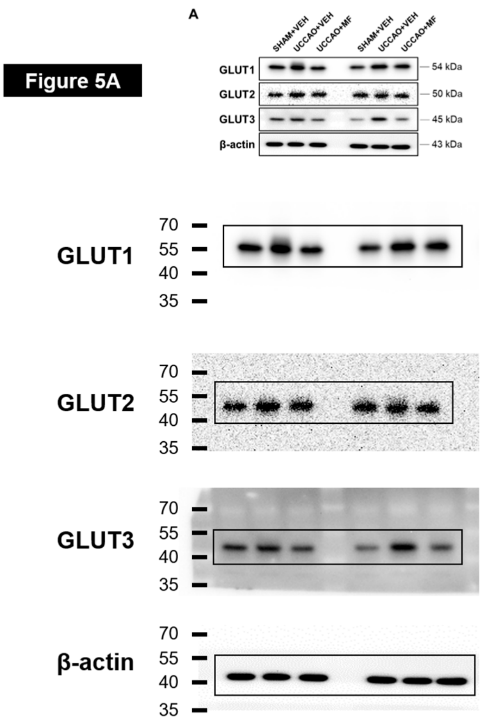

Figure 5B

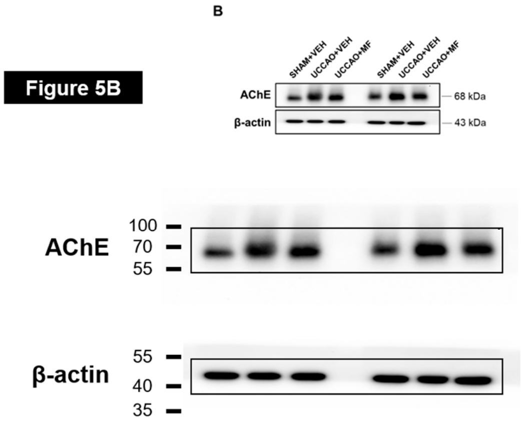

Supplementary Figure S4. Uncropped blots corresponding to Figure 5. Uncropped full-length pictures of Western blotting membranes. The membranes were often cut to enable blotting for multiple antibodies.

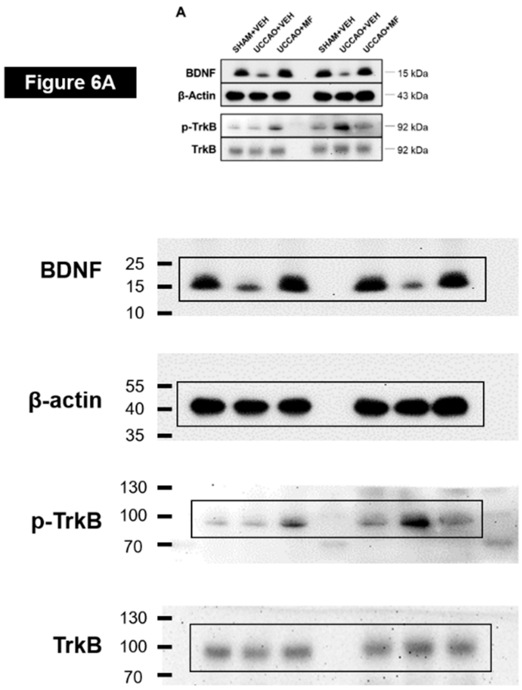

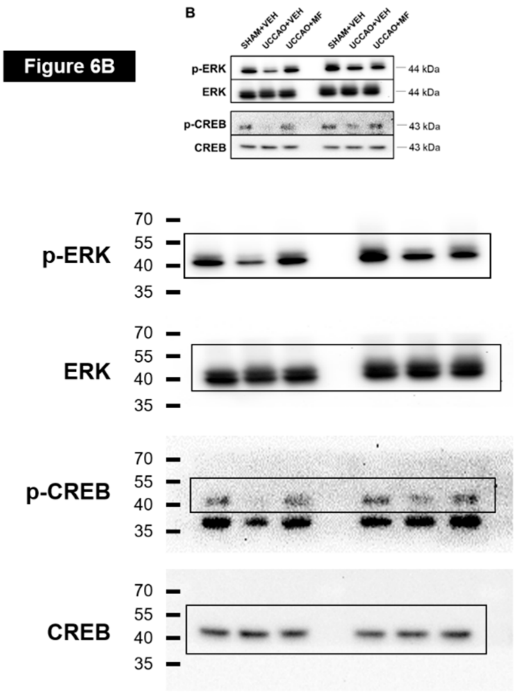

Supplementary Figure S5. Uncropped blots corresponding to Figure 6. Uncropped full-length pictures of Western blotting membranes. The membranes were often cut to enable blotting for multiple antibodies.

Supplementary Table S1. List of primary antibodies used in the present study

Antibody	Company	Catalog number	Dilution
GLUT1	Millipore, USA	07-1401	1:2000
GLUT2	Millipore, USA	07-1402-I	1:1000
GLUT3	Abcam, UK	ab41525	1:1000
AChE	Abcam, UK	ab183591	1:1000
BDNF	Abcam, UK	ab108319	1:1000
p-TrkB	Invitrogen, USA	PA5-36695	1:500
TrkB	Abcam, UK	ab18987	1:500
p-ERK	Cell signaling, USA	9101s	1:1000
ERK	Cell signaling, USA	9102s	1:2000
p-CREB	Cell signaling, USA	9198s	1:1000
CREB	Cell signaling, USA	9197s	1:1000
β-actin	Merch, USA	A5316	1:10000
